# Alcohol acute intoxication before sepsis impairs the wound healing of intestinal anastomosis: rat model of the abdominal trauma patient

**DOI:** 10.1186/1749-7922-7-S1-S10

**Published:** 2012-08-22

**Authors:** Pedro Henrique Alves de Morais, Vinícius Lacerda Ribeiro, Igor Eduardo Caetano de Farias, Luiz Eduardo Almeida Silva, Fabiana Pirani Carneiro, Joel Paulo Russomano Veiga, João Batista de Sousa

**Affiliations:** 1 Medical School, Academic League of Emergency and Trauma, University of Brasilia, Brasilia, Brazil; 2 Medical School, University of Brasilia, Brasilia, Brazil; 3 Campus Universitário Darcy Ribeiro, Prédio da Reitoria, 2º pavimento, sala B2-16, 70910-900 Brasília – DF Brasil, Brazil

## Abstract

**Introduction:**

Most trauma patients are drunk at the time of injury. Up to 2% of traumatized patients develop sepsis, which considerably increases their mortality. Inadequate wound healing of the colonic repair can lead to postoperative complications such as leakage and sepsis.

**Objective:**

To assess the effects of acute alcohol intoxication on colonic anastomosis wound healing in septic rats.

**Methods:**

Thirty six Wistar rats were allocated into two groups: S (induction of sepsis) and AS (alcohol intake before sepsis induction). A colonic anastomosis was performed in all groups. After 1, 3 or 7 days the animals were killed. Weight variations, mortality rate, histopathology and tensile breaking strength of the colonic anastomosis were evaluated.

**Results:**

There was an overall mortality of 4 animals (11.1%), three in the group AS (16.6%) and one in the S group (5.5%). Weight loss occurred in all groups. The colon anastomosis of the AS group didn’t gain strength from the first to the seventh postoperative day. On the histopathological analysis there were no differences in the deposition of collagen or fibroblasts between the groups AS and S.

**Conclusion:**

Alcohol intake increased the mortality rate three times in septic animals. Acute alcohol intoxication delays the acquisition of tensile strength of colonic anastomosis in septic rats. Therefore, acute alcohol intoxication before sepsis leads to worse prognosis in animal models of the abdominal trauma patients.

## Introduction

Abdominal trauma patients are often acutely intoxicated with alcohol, and one of the injuries they can suffer is the rupture of the colon. This injury leads to leakage of feces into the abdominal cavity, and has as consequences peritonitis and sepsis. After surgery, the prognosis of the patient depends to a large extent on the wound healing of the colon.

Healing is a sequential and organized biological process which aims to repair damaged tissue and reunite the edges of the wound, to finally restore both the organ’s physiological functions and the barrier that separates the external and internal environments [1]. It can be divided into four sequential steps: hemostasis, inflammation, proliferation and remodeling [1].

Inadequate wound healing is responsible for postoperative colonic repair complications such as dehiscence and leakage. The postoperative rate of anastomotic leakage in abdominal trauma patients varies from 7% to 14% in low risk patients, and can be as high as 40% in higher risk patients [2]. These complications are responsible for longer hospital stay, reoperation and increased morbidity and mortality [2,3].

Studies have shown that up to 2% of traumatized patients develop sepsis, which considerably increases the mortality if compared to non-septic individuals [4].

Sepsis was the 11th leading cause of death in the U.S. in 2003 and in Brazil the prevalence and mortality are high, with up to 60% of mortality in septic chock [5].

Alcohol is the most consumed drug in the world [6]. Epidemiological data of the emergency units and intervention studies indicate that most patients seen by some traumatic disorder were drunk [7-9].

Over 50% of the beds for trauma are occupied by patients who were acutely intoxicated by alcohol at the time of injury [10]. The intake of alcohol contributes to worsen the injuries caused by trauma and can complicate the management of these patients.

The aim of this study was to assess the impact of acute alcohol intoxication on colonic anastomosis wound healing in rats under sepsis in an experimental model of the abdominal trauma patient.

## Materials and methods

This randomized blinded experimental study was performed after the consent of the Ethics Committee of Animal Usage (CEUA), University of Brasilia. All procedures were guided by ethical standards proposed by the Brazilian College of Animal Experimentation (COBEA).

The study was designed with 36 male Wistar rats, which were randomly allocated into two groups of 18 animals each:

**◊ GROUP S** (**S**epsis): anesthesia, sepsis induction, segmental colectomy, colonic anastomosis, wound healing evaluation.

**◊ GROUP AS** (**A**lcohol and **S**epsis): alcohol intake, anesthesia, sepsis induction, segmental colectomy, colonic anastomosis, wound healing evaluation.

Each group was subdivided into three subgroups of six animals, to be euthanized after 1, 3 or 7 days postoperatively (POD), named as:

**◊ GROUP S:** S1, S3 and S7;

**◊ GROUP AS:** AS1, AS3 and AS7;

On the operation day the rats were fasted for one hour. The animals of the AS group were alcoholized with ethanol diluted in saline to a concentration of 40% with a standard dose of 2 ml of solution. This dose is equivalent to a 480mL spirits intake or approximately 10 shots, in a young adult male of 75kg of weight. Half of the dose (1ml) was administered by mouth, using the gavage method. Another 1ml was given one hour later also by mouth, immediately before anesthesia. The surgeons were blinded to whether the rats had received alcohol or not.

The anesthetic induction was performed with xylazine in a dose of 10 mg / kg, and ketamine at a dose of 75 mg / kg, both intramuscularly. Then the abdomen was cleaned with iodinated detergent.

**Figure 1:**
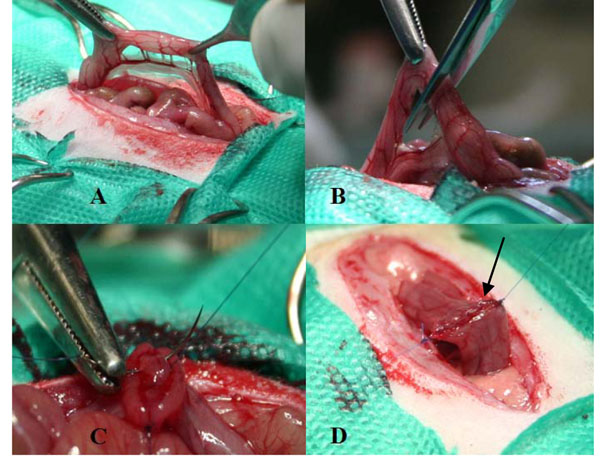
Segmental colectomy and colonic anastomosis in the rat. **A**: identification of the segment of the colon to be resected. **B**: segmental colectomy. **C**: running suture of the posterior anastomotic lip. **D:** colon transit restored by end to end anastomosis, the arrow indicates the suture line.

A midline abdominal incision that began one centimeter cranial to the pubis symphysis, with a length of approximately 4.5 cm, was performed. One centimeter of the left colon was resected, and an end-to-end anastomosis was performed with single layer running sutures, with 6-0 polypropylene (Figure 1). The abdominal wall closure was performed with running sutures, in two layers, using 3-0 polypropylene. Postoperative analgesia was done with tramadol in a dose of 0,72 mg / kg at every 12 hours.

**Figure 2:**
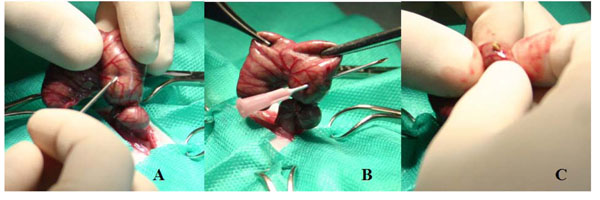
Wichterman sepsis induction method. A and B the cecum is perforated. C the cecum is squeezed to leak feces and induce the sepsis.

Peritonitis was induced, in all groups, by the method of Wichterman *et al. *[11] consisting of a partial ligation of the cecum with cotton suture, immediately below the triangular ileocecal fold to increase the pressure within that segment of the intestine without causing ischemia and allowing free passage of the contents of the small intestine into the large intestine. Then the cecum was perforated in 10 random points with a 40x12mm needle, followed by its compression for fecal leakage (Figure 2).

At 1, 3, or 7 post operative days (POD) the animals were weighed, anesthetized, re-operated and killed with an overdose of thionembutal intravenously.

The anastomotic breaking strength (ABS) was evaluated with a vertical test apparatus called Versa Test (Mecmesin Versa Test, United Kingdom), coupled to a portable digital dynamometer in which the colonic anastomosis samples were attached and pulled with a speed of 25mm/min [12].

The maximum traction force value that the tissue endured before rupture was measured in Newtons.

A sample of the anastomotic scar was collected for histopathological analysis, fixed in formalin and stained by hematoxylin and eosin. The amount of collagen, fibroblast, mononuclear and polymorphonuclear infiltrations and neovascularization were marked with values 0, 1, 2 or 3 each, in which 0 means nothing and 3 a large amount. The parameters of abscess, bacterial colony, foreign body, crust and fibrin were signalized as 0 or 1, meaning absent or present, respectively.

The results were analyzed using SPSS software (Special Package for Social Sciences) version 18.0. Parametric and nonparametric tests were performed, according to the nature of the variables. The paired samples t test was used for the weight variations and Kruskal-Wallis test for anastomotic breaking strength. The Fisher exact test was used to perform the statistical analysis of all histopathological variables. Significance was set at a value of p <0.05.

## Results

**Figure 3:**
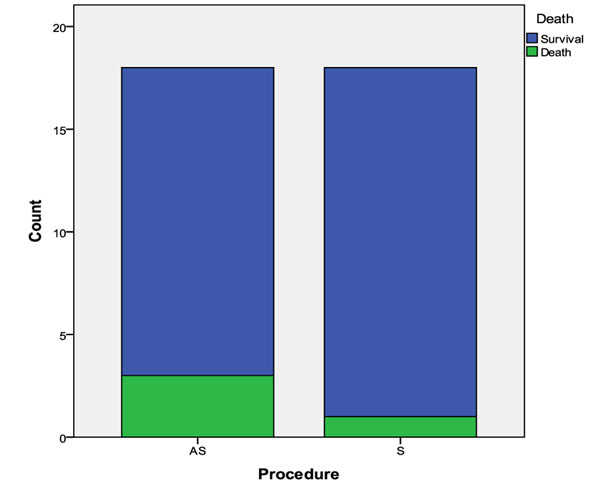
Number of animals that died are in green and those that survived are in blue.

There was an overall mortality of four deaths (11,11%). Three animals from the group AS died (16,6%), one of them in the subgroup AS1 and two in the AS7. In the S group only an animal died, in the S3 group, a death rate of 5,5%. (Figure 3).

There was weight loss in almost every group, from the operation day to the day of euthanize (p < 0,05), as shown in the Table 1. The average preoperative weight of all groups was 321,05 grams, and the post operative weight was 299,6 grams.

**Table 1:** Preoperative and postoperative average weight of each group. The statistically significant differences were signaled.

Weight per group			
	**Preoperative**	**Postoperative**	**P**

**AS1**	320,2	309,7	<0,05*
**AS3**	326,0	291,3	<0,05*
**AS7**	292,6	269,4	<0,05*
**S1**	351,6	348,3	>0,05
**S3**	308,9	272,1	<0,05*
**S7**	313,8	292,6	<0,05*

**Figure 4:**
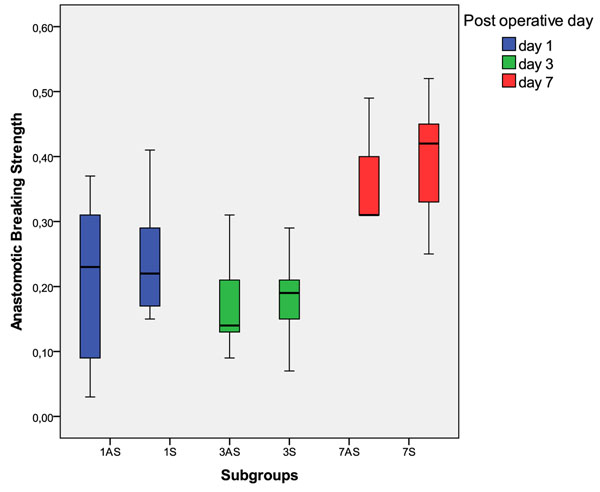
Anastomotic breaking strength distribution in Newtons: superior and inferior limits, interquartils interval and the median in the central part of the boxes. All groups have been displayed.

The anastomotic breaking strength (ABS) was not different between groups AS and S, from the first to the third day (p > 0.05). There was no statistical difference between groups AS1 and S1, AS3 and S3 or AS7 and S7 (p > 0.05), Figure 4 and Table 2.

**Table 2:** Minimum, Maximum, median, mean and standard deviation for the colonic anastomosis breaking strength at each group and subgroups. Values measured in Newtons.

*Anastomosis Breaking Strength*						
	**AS1**	**S1**	**AS3**	**S3**	**AS7**	**S7**

**n (survived)**	5	6	6	5	4	6
**Minimum**	0,03	0,15	0,09	0,07	0,31	0,25
**Maximum**	0,37	0,41	0,31	0,29	0,49	0,52
**Median**	0,23	0,22	0,14	0,19	0,31	0,42
**Mean**	0,20	0,24	0,17	0,18	0,35	0,40
**Std. Deviation**	0,14	0,09	0,08	0,08	0,09	0,09

**Figure 5:**
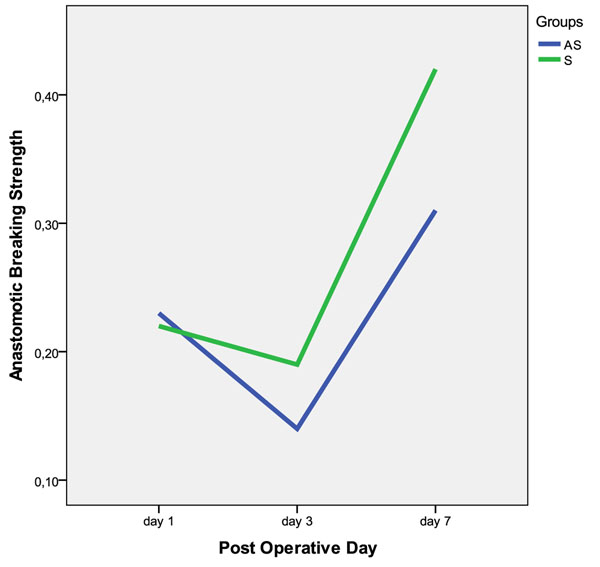
Median of the anastomotic breaking strength, in Newton, one, three and seven post-operative days. Even after 7 days the AS group (blue) colonic anastomosis did not become stronger (p>0,05) while the S group (green) did (p<0,05).

There was no difference between the groups AS1, AS3 and AS7 (p > 0,05). The S7 group had a higher anastomotic breaking strength than S1 and S3 (p < 0,05). The AS group anastomotic breaking strength (ABS) had a sharper decline than the S group from the first to the third day, and at the seventh day the AS group ABS was not recovered (Figure 5).

On the histopathological evaluation there was no difference of any of the analyzed parameters between groups AS and S (Table 3). There was no difference of collagen between the groups AS7 and S7 (p>0.05).

**Table 3:** Sum of the values of all animals of the groups due to the analyzed histopathological parameters. The amount of collagen, fibroblast, mononuclear and polymorphonuclear infiltrations and the neovascularization were marked with values from 0 to 3 each, in which 0 means nothing and 3 a large amount. The parameters of abscess, bacterial colony, foreign body, crust and fibrin were signalized as 0 or 1, meaning absent or present respectively.

Histopathological Analysis						
	**AS1**	**S1**	**AS3**	**S3**	**AS7**	**S7**
	n=5	n=6	n=6	n=5	n=4	n=6

**Collagen**	0	0	0	0	4	6
**Fibroblast**	0	0	6	5	12	18
**Neovascularization**	0	0	6	5	8	12
**Mononuclear**	0	0	6	5	8	12
**Polymorphonuclear**	7	6	15	13	9	13
**Abscess**	1	0	4	4	1	1
**Bacterial Colony**	1	1	2	5	2	1
**Foreign Body**	1	0	2	3	4	5
**Crust**	5	6	4	5	3	4
**Fibrin**	4	5	6	5	0	0

## Discussion

The aim of this study was to evaluate the effects of acute ethanol exposure at single high dose just before an injury in rats with fecal sepsis. To evaluate that, we have analyzed the death rate, weight variations, anastomosis breaking strength and histopathology.

Both alcohol and sepsis are known to lead to weight loss after surgery, and their combination diminished the post-operative body mass in this study, and even at the 7 POD that weight wasn’t recovered [13,14]. Sepsis leads to a consumptive syndrome due to the inflammation and alcohol intake is responsible for malnutrition because of intestinal malabsorption and is also responsible for body fat reduction [13,14].

Sepsis is an important cause of death in trauma patients. It was the cause of 9% of deaths in a level I trauma centre in USA in 2003 [15]. Alcohol is also a risk factor for death in animal models and human patients [13,16,17]. This study showed that the combination of alcohol and sepsis have an even greater impact on postoperative mortality, since the group AS had a death rate three times greater the S group.

The scar tissue healing can be mechanically evaluated by both longitudinal anastomotic breaking strength (ABS) used in our study and radial bursting strength [13,18]. Longitudinal breaking strength is the measure of intestinal wall resistance to forces applied on its longitudinal direction while bursting pressure measures the resistance to intraluminal elevated preassures [19]. The ABS measures the risk for total dehiscence, because it measures the strength of the scar’s strongest bit, while the bursting pressure evaluates the risk of leakage, because it measures the strength of the weakest segment of the scar.

The AS group colon surgical wound didn’t became stronger by day 7, because it was not different from the 3AS or the 1AS groups (p> 0,05). The acquisition of tensile strength of the wound is due to the deposition and organization of the collagen, and an impaired wound healing is responsible not only for the lack of collagen, but also for disorganized collagen [1]. It is possible that the alcohol intake was responsible for an impaired inflammation stage of the wound healing and magnified the deleterious effects of sepsis, such as disorganized deposition of collagen and excessive activity of matrix metalloproteases [1,20-22].

The effects of alcohol on wound healing are dependent to the pattern of the alcohol exposure: chronic or acute abuse, the dose intake, duration of consumption, time from alcohol exposure to injury, alcohol withdrawal and associated factors such as infection, sepsis, smoking, usage of medication, obesity, diabetes, and other comorbidities [1]. Acute ethanol exposure in non-septic patients can lead to inadequate wound healing, by impairing the early inflammatory response, inhibiting wound closure, angiogenesis and collagen production, and changing the protease balance at the wound site [1], although we didn’t observe this in the septic conditions of this study.

Inflammation is a normal part of the wound healing process, and is important to the removal of contaminating micro-organisms [1]. In the absence of effective decontamination, such as in fecal sepsis, inflammation may be prolonged, thus the next steps in wound healing, the inflammation and remodeling, can be prolonged or impaired, but not always [1]. Both bacteria and endotoxins can lead to prolonged elevation of pro-inflammatory cytokines such as interleukin-1 (IL-1), IL-6, IL-10, TNF-α, and increased levels of matrix metalloproteases (MMP) [1,20-22].

## Conclusions

Sepsis and its association with ethanol led to weight loss postoperatively. Alcohol intake increased the mortality rate three times in septic animals. Acute alcohol intoxication delays the acquisition of tensile strength of colonic anastomosis in septic rats. Therefore, acute alcohol intoxication before sepsis leads to worse prognosis in animal models of abdominal trauma patients.

## Competing interests

The authors declare that they have no competing interests of any kind.

## Authors’ contributions

PHAM did the project design and coordination, surgical and technical work, statistical analysis, data acquisition and interpretation and manuscript writing. VLR, IECF and LEAS all did the project design, surgical and technical work, data acquisition and interpretation. FPC was responsible for the histopathological work and data interpretation. JPRV helped with the project design, technical work and data interpretation. JBS did the data interpretation, critical review and manuscript writing. All authors read and approved the final manuscript.
